# CSF macrophage migration inhibitory factor levels did not predict steroid treatment response after optic neuritis in patients with multiple sclerosis

**DOI:** 10.1371/journal.pone.0207726

**Published:** 2018-11-26

**Authors:** Marc Pawlitzki, Catherine M. Sweeney-Reed, Sven G. Meuth, Dirk Reinhold, Jens Neumann

**Affiliations:** 1 Department of Neurology, Otto-von-Guericke University, Magdeburg, Germany; 2 Department of Neurology with Institute of Translational Neurology, University Hospital Muenster, Muenster, Germany; 3 Institute of Molecular and Clinical Immunology, Otto-von-Guericke-University, Magdeburg, Germany; Julius-Maximilians-Universität Würzburg, GERMANY

## Abstract

Glucocorticoid (GC) refractory relapses in patients with multiple sclerosis (MS) or clinically isolated syndrome (CIS), who are in potential need of treatment escalation, are a key challenge in routine clinical practice. The pro-inflammatory cytokine macrophage migration inhibitory factor (MIF) has been shown to be an endogenous counter-regulator of GC, and potentiates autoimmune-mediated neuroinflammation. In order to evaluate whether MIF levels are elevated in the cerebrospinal fluid (CSF) of MS patients (CSF-MIF), and whether they are higher still during a GC refractory relapse, we compared CSF-MIF concentrations of CIS/MS patients with acute optic neuritis as their first inflammatory episode (ON, n = 20), CIS/MS patients with a stable disease progression/without relapse (CIS/MS w/o, n = 18), and healthy controls (HC, n = 20) using ANOVA. Mean CSF-MIF concentrations in CIS/MS w/o patients were significantly higher than in ON patients and HCs, whereas ON patients and HCs did not differ. A subgroup analysis of the ON group revealed 10 patients to be responsive to GC-treatment (GC-ON) and 10 patients refractory under GC-treatment (rGC-ON). However, mean CSF-MIF concentrations did not differ between GC-ON and rGC-ON cases. We therefore conclude that MIF is not suitable for distinguishing GC responders from non-responders in a group of patients with acute optic neuritis, but it rather mirrors the ongoing inflammation in long-term MS disease progression.

## Introduction

Acute inflammatory relapses are the main hallmark of persisting and ongoing disease-related disability in patients with multiple sclerosis (MS) [1]. Great advances of disease-modifying therapies to reduce annual relapse rates have been made. However, the treatment of acute relapses with intravenous high-dose glucocorticoid (GC) administration has remained largely unaltered [2], even though severe, steroid-refractory relapses are common in clinical routine [3] and not well-understood. The treatment escalation for steroid-refractory relapses includes therapeutic apheresis or immunoadsorption, which have shown promising results, leading to the question of whether such treatment options should be started earlier or even replace steroid therapy [4–6]. MS is characterized by different inflammatory cascades and changing levels of proinflammatory factors [7,8], which might be the reason why some relapses are responsive to GC-treatment, while others are refractory. These features of MS stand in contrast to other neurological disorders, such as autoimmune-related diseases, where specific humoral factors–in particular autoantibodies–have been recognized [9–11], leading to more established treatment strategies for these disorders. Similar to the experiences made with disease-modifying therapies, the individual responses to immunosuppression/-modulation differ significantly between individual MS cases, rendering the identification of potential biomarkers an important challenge.

Macrophage migration inhibitory factor (MIF) is a pro-inflammatory cytokine, which is required for antigen-driven T-cell activation and sustained inflammation in autoimmune disorders [12,13]. In addition, MIF exhibits an endogenous counter-regulation of the anti-inflammatory effects of GCs [14–16] and leads to a potentially decreased response to GC-treatment in autoimmune diseases [17–20]. Evidence indeed suggests that MIF mediates GC resistance [21]. The inhibition of MIF has been shown to act synergistically with GC-treatment in experimental autoimmune encephalomyelitis (EAE), an established animal model of MS, with the potential of providing a steroid sparing treatment approach [19,22]. Elevated MIF levels in the blood and cerebrospinal fluid (CSF) of MS patients have recently been reported and were found to be related to clinical progression [23,24], suggesting that MIF levels could potentially serve as a biomarker in MS.

We hypothesized that elevated CSF-MIF concentrations are related to an acute inflammatory event in CIS/MS and to steroid-refractory relapses. Moreover, we postulated that MIF can potentially provide a valuable biomarker for the earlier initiation of plasma separation techniques as an alternative to GC-treatment.

## Material and methods

### Patients, controls and clinical assessment

Our pilot study involved measuring MIF-concentrations in the diagnostically obtained CSF from 20 patients with optic neuritis (ON) as first inflammatory episode in clinically isolated syndrome (CIS, n = 9) or relapsing-remitting MS (RRMS, n = 11), according to the McDonald criteria (2010) [25]. The second group of CSF-MIF measurements were from 18 patients who had been diagnosed with CIS (n = 9) / MS (n = 9) but had not presented with an acute clinical relapse within the preceding 4 weeks or received any steroids within the 8 weeks (CIS/MS without acute relapse = CIS/MS w/o) prior to study inclusion (mean [SD] latency between last relapse and lumbar puncture = 31 [39] months). CSF was additionally acquired from a hospital-based cohort of n = 20 healthy controls (HC). In these healthy individuals the presence of a neurological disorder had been suspected, but was not confirmed. In addition to the clinical classification, patients included in the control group also fulfilled the following Reiber laboratory criteria defining a non-inflammatory CSF (< 5 cells/μl, > 500 mg protein/ml, < 2 mmol/l lactate, no disruption of the blood/CSF barrier, no oligoclonal bands (OCB) in the CSF, and no intrathecal immunoglobulin (Ig) G, IgA, or IgM synthesis) [26]. Patients of all three groups were clinically evaluated in accordance with German guidelines (including the Expanded disability status scale [EDSS] [27]). Disease duration was defined as the time in months between symptom onset and lumbar puncture (LP). No patient had received any disease-modifying treatment before LP.

In the ON group, visual acuity was measured preceding GC-treatment, on the last day of GC administration, as well as after therapy escalation including plasma exchange. To verify ON-induced axonal disintegration, visual evoked potentials (VEP) were recorded before GC-treatment in at least two trials for each eye, averaging >150 responses, with electrodes positioned at Oz (active) and Fz, in accordance with the International Society for Clinical Electrophysiology of Vision (ISCEV) standards [28]. Latencies of the P100 exceeding a 2.5 standard deviation (SD) from normative data were considered as abnormal VEPs. P100 peak-latencies were measured to reflect the degree of demyelination. VEPs with no stimulation response were excluded (n = 6/20). Brain MRI scans from patients originated from non-standardized protocols from differing MRI units and magnetic field strengths (1.5 or 3.0 Tesla) conducted close to the LP. All examinations included T1- and T2-weighted spin-echo sequences with the administration of gadolinium (Gd). Abnormalities including T1-hypointesities, T2-hyperintesities and Gd-enhanced T1-lesions were initially identified by a neuroradiologist and they were subsequently verified by a MS specialist (M.P.).

Irrespective of the degree of relapse-induced visual loss, ON patients were treated for at least 5 days with a high daily dose (1 g) of intravenous methylprednisolone. For this group, GC-responsive ON (GC-ON) was defined as visual improvement after 5 days of treatment, with a visual acuity correction of >10% compared to baseline, whereas GC-refractory ON (rGC-ON) was defined by a 0–10% change in visual acuity.

All patients were recruited retrospectively in the Department of Neurology, Otto-von-Guericke University, Magdeburg, Germany, between 2012 and May 2017. Thus, no written informant consent could be obtained, but all parameters were taken from routine clinical diagnostics. The data were anonymized, precluding identification of individual patients. The study was approved by the Local Ethics Committee of the Faculty of Medicine at the University Hospital Magdeburg and Otto-von-Guericke University Magdeburg (No. 07/17).

### MIF measurement

Immediately after LP, CSF cell concentration was determined, and total protein, albumin quotient (Qalb), and OCB were measured. The remaining CSF material was centrifuged at 4°C, aliquoted, and stored at -80°C until CSF-MIF analysis took place. The absolute CSF-MIF concentrations were measured using commercially available human MIF Quantikine ELISA (bio-techne, Mineapolis, MN), following the instructions provided by the manufacturer. All samples were run in duplicate, and the mean was used for statistical analysis.

### Statistical analysis

Statistical analysis was conducted using SPSS 21 (IBM). A one-way-ANOVA was conducted with *group* (HC vs. CIS/MS w/o vs. ON cases) as the independent variable, followed by pairwise post-hoc testing (Bonferroni-corrected).

The groups were also compared with respect to categorical variables (using a chi-squared test) and continuous variables (using a t-test or Mann-Whitney U test), to determine group differences between GC-ON and rGC-ON cases, taking into account age, median visual acuity before and after GC administration, P100 latency, CSF cell count, Q_alb_, and CSF-MIF. A Wilcoxon test was used to determine whether visual improvement occurred after plasma exchange in the rGC-ON group.

Spearman’s rank correlations were performed between CSF-MIF and age, further CSF measures (cell count, Q_alb,_ relative monocyte fraction), disease duration, T2-lesion count and P100 latency. P-values ≤ 0.05 were deemed to be statistically significant.

## Results

### Comparison between ON patients, CIS/MS w/o, and HC

The demographical, clinical, and CSF data of the cohorts are shown in **Table 1.** We did not observe significant differences in age, sex, CSF protein, or Q_alb_ between ON cases, CIS/MS w/o, and HCs, whereas CSF cell counts varied between the groups **(Table 1).** As expected, the mean [SD] disease duration of CIS/MS w/o cases was significantly longer than that in the ON group (940 [1189] vs. 7 [6] days).

**Table 1:** 

	HC (N = 20)	CIS/MS w/o (N = 18)	ON (N = 20)	p-values		
				HC vs. CIS/MS w/o	HC vs. ON	CIS/MS w/o vs. ON
**Age at lumbar puncture (years)**	33 [9] (17–46)	37 [13] (17–61)	31 [9] (17–48)	0.6	1.0	0.3
**Female sex, N (%)**	14 (70)	14 (78)	14 (70)	0.8	0.8	0.8
**Disease duration (days)**	-	940 [1189] (34–3722)	7 [6] (0–25)	-	-	**0.006**
**Median EDSS (range)**	-	1.5 (0–3.5)	3 (1.5–4)			**< 0.001**
**Cerebral T2 lesion count**	-	8 [9.0] (2–39)	6 [4.5] (1–19)			0.4
**CSF Cell count /μl**	2 [1] (0–4)	7 [6] (1–25)	9 [9] (0–35)	**0.02**	**0.002**	1.0
**CSF protein (mg/dl)**	324 [65] (178–460)	393 [118] (255–650)	370 [135] (234–825)	0.2	0.6	1.0
**Positive OCB, N (%)**	0	18 (100)	20 (100)	-	-	1.0
**Qalb**	4.3 [1.1] (2.3–6.4)	4.9 [1.8] (2.5–8.9)	4.7 [2.2] (2.6–11.5)	0.9	1.0	1.0
**Rel. monocyte fraction**	0.30 [0.15] (0.12–0.68)	0.22 [0.20] (0.02–0.62)	0.18 [0.20] (0.03–0.80)	0.6	0.1	1.0
**CSF-MIF**	8.6 [2.7] (2.0–14.1)	10.9 [2.8] (6.3–16.6)	8.6 [2.8] (4.0–13.4)	**0.04**	1.0	**0.03**

N = number of participants; unless otherwise reported mean [standard deviation] (range) is given. CSF = cerebrospinal fluid, EDSS = Expanded disability status scale, OCB = oligoclonal bands, HC = healthy controls, MIF = Macrophage migration inhibitory factor, MS w/o = Clinically isolated syndrome or multiple sclerosis without an acute relapse, ON = acute optic neuritis group, Qalb = albumin quotient. Rel. = relative. Disease duration was defined as the timespan between symptom onset and the date of lumbar puncture. For group comparisons a chi-squared test or an ANOVA analysis of variance with post-hoc Bonferroni-testing were conducted. P-values ≤ 0.05 were deemed to be statistically significant.

**Fig 1:**
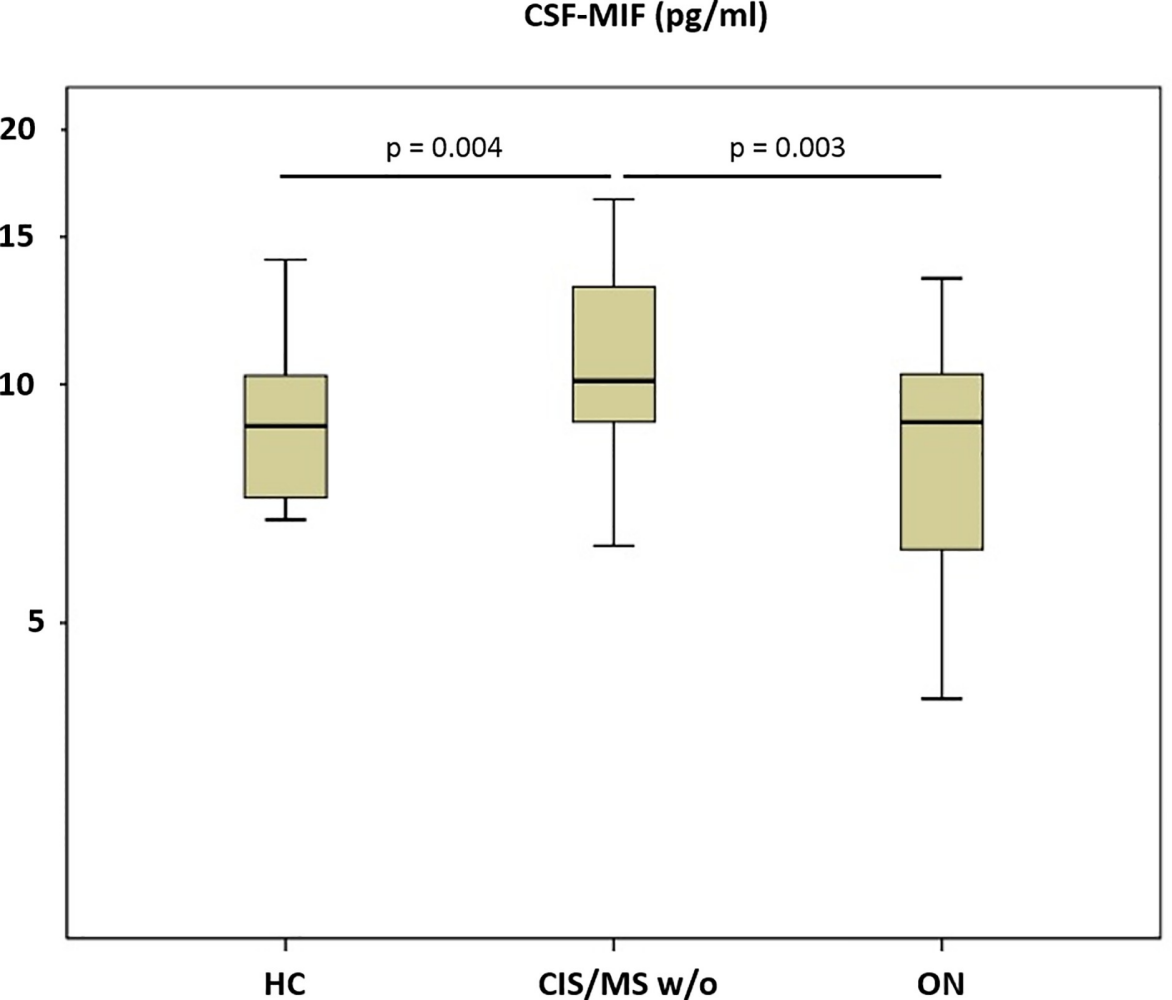
Macrophage migration inhibitory factor (MIF) in cerebrospinal fluid (CSF). HC = healthy controls. CIS/MS w/o = Clinically isolated syndrome or multiple sclerosis without an acute relapse, ON = optic neuritis. Boxes indicates the interquartile range, bars indicates median CSF-MIF values, and whiskers present the 95% CI. Group comparisons were conducted using an ANOVA with post-hoc Bonferroni-testing. P-values ≤ 0.05 were deemed to be statistically significant. MS w/o patients showed higher CSF-MIF levels than HC and ON patients, while the latter did not differ.

There was a significant effect of group on CSF-MIF (p = 0.02), and post-hoc analysis revealed higher mean [SD] CSF-MIF levels in MS w/o (10.9 [2.8] pg/ml) than in the ON (8.6 [2.8] pg/ml) and HC groups (8.6 [2.7] pg/ml), while the latter two groups showed no difference (**Fig 1**).

### Comparison between GC-ON and rGC-ON

Out of 20 patients with acute ON, 10 cases were categorized as GC-ON or rGC-ON, respectively. Three out of 10 patients in the GC-group and 8 out of 10 patients in the rGC-ON group met the criteria for MS. Mean [SD] age (32 [11] vs. 30 [7]) did not differ, while sex distribution was significantly different between both groups (100% vs. 40%, p = 0.03).

The mean [SD] time between the onset of ON and steroid administration was balanced (10 [7] days vs. 5 [4] days). Eight rGC-ON cases were treated with plasma exchange, with treatment onset averaging at 5 days after the last GC administration. Two rGC-ON cases received methylprednisolone only, and no plasma exchange was performed. Prior to the first GC administration, median [range] visual acuity was significantly lower in the rGC-ON group (0.05 [0–0.3]) compared to the GC-ON cases (0.2 [0.1–0.8]) and remained significantly lower after GC administration (0.05 [0–0.4] vs. 0.69 [0.5–1.0]). Moreover, mean [SD] P100 latency was significantly higher in rGC-ON cases (142 [17] ms vs. 123 [9]) **(Table 2).** Within the rGC-ON group, median visual acuity markedly increased in 8 of 10 patients after therapy escalation. Despite the clinical and diagnostic difference between both groups (visual acuity, P100 latency), the mean [SD] concentration of MIF in the CSF showed no significant statistical difference (rGC-ON: 8.9 [2.7] pg/ml vs. GC-ON: 8.4 [2.5] pg/ml).

**Table 2:** 

	GC-ON (N = 10)	rGC-ON (N = 10)	p-values
			GC-ON vs. rGC-ON
**Age at lumbar puncture (years)**	32 [11] (18–49)	30 [7] (25–71)	0.6
**Female sex, N (%)**	10 (100)	4 (40)	0.03
**Median EDSS (range)**	2 (1.5–4)	3 (2–4)	0.08
**Cerebral T2-lesion count**	4.5 [3.4] (1–11)	8.0 [5.1] (3–19)	0.09
**Timespan between acute symptom and steroid application (days)**	10 [7] (3–24)	5 [4] (0–14)	0.09
**Median baseline VA (range)**	0.2 (0.1–0.8)	0.05 (0–0.3)	0.004
**Median VA after treatment (range)**	0.69 (0.5–1.0)	0.05 (0–0.4)	**< 0.001**
**Pathological VEP, N (%)**	10 (100)	10 (100)	1.0
**P 100 latency (ms), N**	123 [9] (113–138), 8/10	142 [17] (113–163), 6/10	0.02
** CSF Cell count/μl**	8 [8] (0–23)	10 [10] (1–35)	0.6
** CSF protein (mg/dl)**	325 [55] (234–402)	420 [180] (250–825)	0.1
** Qalb**	3.9 [0.9] (2.6–5.4)	5.7 [3] (3.2–11.5)	0.09
** Rel. monocyte fraction**	0.13 [0.25] (0.03–0.25)	0.22 [0.26] (0.05–0.80)	0.3
** CSF-MIF**	8.4 [2.5] (4.0–12.2)	8.9 [2.7] (5.3–13.4)	0.7

N = number of participants; unless otherwise reported mean [standard deviation] (range) is given. CON = disease controls, CSF = cerebrospinal fluid, MIF = Macrophage migration inhibitory factor, EDSS = Expanded disability status scale, GC-ON = glucocorticoid responsive optic neuritis, Qalb = albumin quotient, Rel. = relative, rGC-ON = non-glucocorticoid-responsive optic neuritis, VA = visual acuity. Disease duration was defined as the timespan between symptom onset and the date of lumbar puncture. Mean P100 latency only could be estimated from patients with stimulation response. For continuous variables, an independent-samples t test or a Mann-Whitney-U test was conducted, while for binary variables, a chi-squared test was calculated. P-values ≤ 0.05 were deemed to be statistically significant.

### MIF correlations between demographic, clinical, and CSF parameters

When including the whole patient sample, correlations with a small to medium effect size were found between CSF-MIF concentration and disease duration (rho = 0.4, p = 0.03), Q_alb_ (rho = 0.5, p = 0.002), and CSF protein (rho = 0.4, p = 0.03), while there was no correlation with sex (Z = -0.2, p = 0.8), CSF cell count (rho = 0.2, p = 0.3), relative fraction of monocytes (rho = 0.1, p = 0.3), EDSS (rho = -0.2, p = 0.3), T2-lesion count (rho = 0.04, p = 0.9) or VEP P100 latency (rho = 0.2, p = 0.6).

## Discussion

This study was designed to investigate the levels of the proinflammatory cytokine MIF in the CSF of MS patients (i) with acute optic neuritis (ON) and (ii) in the absence of an acute relapse. Furthermore, we questioned whether MIF levels might indicate GC responsiveness in patients with acute optic neuritis.

MIF has been found to play a role in a diverse range of physiological as well as pathological processes [29], so does not provide a specific physicochemical biomarker for MS-related disease processes, in particular MIF blood concentration [14]. Indeed, MIF may be secreted by a wide range of cells, including macrophages, T-cells, dendritic, endothelial, and epithelial cells [30–32] and it is constitutively expressed in cerebral and spinal neurons as well as in microglia [33,34]. Release and detection of MIF reveals activity pertaining to inflammation, chemotaxis, cell survival and proliferation [14,15,35]. Similar to basal MIF levels in sera, MIF can also be detected in non-inflammatory brains, but is greatly increased following inflammatory stimuli [36,37]. In MS, perivascular accumulation of inflammatory cells in the CNS following demyelination and microglial cell activation might lead to higher MIF levels in the CSF [19,23].

In addition to its key role in diverse inflammatory processes, MIF may be secreted by tumors [30,36], and its potential as a (tumor) biomarker has also been investigated [35,37].

The processes of MIF secretion are not well-understood, because of its non-classical secretory pathway [30,32]. Evidence has been found that when MIF is secreted, it is initially stored in vesicles in certain cell types, prior to its release via an ATP-binding cassette transporter [32,36]. The half-life of MIF depends on the cells from which it is secreted. The half-life of MIF secreted by normal prostate cells has been reported as 9 h, with an increased stability when secreted from prostate cancer cells resulting in a half-life of 36 h [37]. The MIF protein half-life in normal human cervical epithelial cells has been reported to exceed 18 h [38]. Cell MIF mRNA protein half-life has been found to be approximately 7 h in human endometrial cells [39] and around 19 h in normal human prostate cells [37]. To our knowledge, the half-life of serum and CSF MIF in patients with MS has not yet been investigated. Inference from the half-life reported elsewhere, in combination with the duration of symptoms in MS, it is unlikely that MIF levels fluctuate rapidly, and we do not expect that the stability of MIF will have influenced our findings. Future work should, however, investigate the serum and CSF half-life of MIF in this patient group.

Furthermore, MIF is capable of overcoming the immune suppressive effects of GC at a transcriptional and post-transcriptional level by overriding the GC-induced MAPK-phosphatase-1 expression and inhibiting cytokine production [20,40]. Apart from its physiological role as a counter-regulator of GC effects, elevated MIF expression seems to play a major role in several autoimmune disorders, including rheumatoid arthritis and autoimmune hepatitis [12,17,18], and renders anti-MIF treatment [41,42] a promising therapeutic strategy. This approach has already been successfully demonstrated in experimental models of MS [22].

GCs are frequently used to treat relapses in MS [2]. While the vast majority of patients benefit from GC-treatment, and recover almost completely, the remaining patients do not respond, and the neurological deficits persist. MIF, with its ability to counteract GC activity, might be a component in GC resistance in some patients, leading to the necessity of applying higher doses with, unfortunately, severe side-effects [43].

Based on the elevated CSF-MIF levels in MS patients [23], we hypothesized that higher CSF-MIF levels would be found in patients during a relapse that shows no improvement after treatment with GCs. Our analysis involving patients with ON, however, did not elicit differences in CSF-MIF levels between GC responders and non-responders.

We found significantly elevated CSF-MIF levels in MS patients without relapse activity compared to ON cases and healthy controls. Although these patients had been recently diagnosed with MS, their medical histories revealed several neurological deficits defined as demyelinating events in the past, such that the mean disease duration across patients was estimated to be approximately three years. The generally elevated CSF-MIF levels in these untreated MS patients could potentially be explained by the extended disease duration, in line with the positive correlation we found between MIF levels and disease duration, as well as with previous findings [23]. Moreover, this interpretation would fit with the higher burden of MS-associated inflammatory lesions, in which an increased expression of the MIF receptor CD74 has been detected [44], and ongoing diffused inflammation [45], in contrast to the short mean disease duration (7 days) across the ON-group.

Here, we selected ON patients with the advantage of objectively assessing the outcome using visual acuity and VEP measures, and thus the response to GCs.

However, ON provokes a localized, well-circumscribed inflammation at a large distance from the lumbar CSF, and accordingly might not trigger the release of sufficient amounts of MIF or other cytokines into the CSF [46,47]. In contrast, the appearance of myelitis could lead to a pronounced inflammation, and in turn, yield higher concentrations of MIF in the CSF [23] due to spinal microglia activation [48]. This issue could be addressed in future investigations. We report here the findings from a pilot study. Limitations include the relatively small sample size and the retrospective design. In conclusion, MIF might not suitable for distinguishing GC responders from non-responders in optic neuritis.

## Supporting information

S1 Table CSF *=* cerebrospinal fluid, EDSS *=* Expanded disability status scale, HC *=* healthy controls, GC-ON *=* glucocorticoid responsive optic neuritis, MIF *=* Macrophage migration inhibitory factor, MS w/o *=* Clinically isolated syndrome or multiple sclerosis without an acute relapse, ON *=* acute optic neuritis group, rGC-ON *=* non-glucocorticoid-responsive optic neuritis. (DOCX)
