# Supplementary material for: CSF macrophage migration inhibitory factor levels did not predict steroid treatment response after optic neuritis in patients with multiple sclerosis
Source: PLoS One. 2018 Nov 26;13(11):e0207726. doi: 10.1371/journal.pone.0207726 (PMC6261107; doi:10.1371/journal.pone.0207726)
Supplement: S1 Table — (DOCX) [file pone.0207726.s001.docx]

**Supplementary table 1**

| ID | sex | Age (years) | group | ON-group | CSF-MIF | EDSS |
| --- | --- | --- | --- | --- | --- | --- |
| 1 | female | 17 | CIS/MS w/o |  | 6.3 | 1 |
| 2 | female | 32 | CIS/MS w/o |  | 8.1 | 3 |
| 3 | female | 50 | CIS/MS w/o |  | 8.1 | 2 |
| 4 | female | 48 | CIS/MS w/o |  | 8.2 | 3.5 |
| 5 | female | 26 | CIS/MS w/o |  | 9 | 1 |
| 6 | female | 21 | CIS/MS w/o |  | 9.3 | 1 |
| 7 | female | 49 | CIS/MS w/o |  | 9.4 | 1 |
| 8 | male | 33 | CIS/MS w/o |  | 9.4 | 3 |
| 9 | female | 29 | CIS/MS w/o |  | 9.9 | 1.5 |
| 10 | female | 36 | CIS/MS w/o |  | 10.3 | 0 |
| 11 | female | 47 | CIS/MS w/o |  | 11.1 | 2 |
| 12 | female | 47 | CIS/MS w/o |  | 11.6 | 2 |
| 13 | female | 21 | CIS/MS w/o |  | 12.7 | 1 |
| 14 | male | 26 | CIS/MS w/o |  | 13.1 | 1.5 |
| 15 | female | 32 | CIS/MS w/o |  | 13.3 | 1 |
| 16 | female | 58 | CIS/MS w/o |  | 14.3 | 2 |
| 17 | male | 34 | CIS/MS w/o |  | 15.5 | 2 |
| 18 | female | 61 | CIS/MS w/o |  | 16.6 | 0 |
| 19 | female | 25 | HC |  | 2 |  |
| 20 | female | 22 | HC |  | 4 |  |
| 21 | male | 35 | HC |  | 6.8 |  |
| 22 | female | 31 | HC |  | 7.2 |  |
| 23 | female | 20 | HC |  | 7.2 |  |
| 24 | female | 33 | HC |  | 7.3 |  |
| 25 | male | 31 | HC |  | 7.5 |  |
| 26 | female | 32 | HC |  | 7.6 |  |
| 27 | male | 17 | HC |  | 8.3 |  |
| 28 | male | 46 | HC |  | 8.6 |  |
| 29 | male | 38 | HC |  | 9.2 |  |
| 30 | male | 43 | HC |  | 9.3 |  |
| 31 | female | 29 | HC |  | 9.8 |  |
| 32 | female | 18 | HC |  | 10 |  |
| 33 | female | 34 | HC |  | 10.2 |  |
| 34 | female | 37 | HC |  | 10.3 |  |
| 35 | female | 34 | HC |  | 10.3 |  |
| 36 | female | 45 | HC |  | 11.7 |  |
| 37 | female | 46 | HC |  | 11.7 |  |
| 38 | female | 39 | HC |  | 14.1 |  |
| 39 | female | 46 | ON | GC-ON | 395 | 1.5 |
| 40 | female | 43 | ON | GC-ON | 616 | 2 |
| 41 | female | 33 | ON | GC-ON | 6.3 | 2 |
| 42 | female | 36 | ON | GC-ON | 7.7 | 3.5 |
| 43 | female | 22 | ON | GC-ON | 7.8 | 3 |
| 44 | female | 28 | ON | GC-ON | 9.4 | 2 |
| 45 | female | 48 | ON | GC-ON | 9.7 | 2 |
| 49 | female | 27 | ON | GC-ON | 9.7 | 3 |
| 47 | female | 18 | ON | GC-ON | 10.7 | 4 |
| 48 | female | 23 | ON | GC-ON | 12.2 | 2 |
| 49 | female | 35 | ON | rGC-ON | 5.3 | 3 |
| 50 | male | 27 | ON | rGC-ON | 5.85 | 3 |
| 51 | female | 33 | ON | rGC-ON | 5.93 | 3 |
| 52 | male | 28 | ON | rGC-ON | 7.5 | 2 |
| 53 | male | 34 | ON | rGC-ON | 8.6 | 3.5 |
| 54 | female | 41 | ON | rGC-ON | 9.9 | 4 |
| 55 | male | 31 | ON | rGC-ON | 9.9 | 3 |
| 56 | female | 17 | ON | rGC-ON | 10.7 | 3.5 |
| 57 | male | 22 | ON | rGC-ON | 11.5 | 3 |
| 58 | male | 35 | ON | rGC-ON | 13.4 | 3 |

Supplementary table: CSF = cerebrospinal fluid, EDSS = Expanded disability status scale, HC = healthy controls, GC-ON = glucocorticoid responsive optic neuritis, MIF = Macrophage migration inhibitory factor, MS w/o = Clinically isolated syndrome or multiple sclerosis without an acute relapse, ON = acute optic neuritis group, rGC-ON = non-glucocorticoid-responsive optic neuritis
